# High-Voltage Polyanion Positive Electrode Materials

**DOI:** 10.3390/molecules26175143

**Published:** 2021-08-25

**Authors:** Atsuo Yamada

**Affiliations:** Department of Chemical System Engineering, The University of Tokyo, Tokyo 113-8656, Japan; yamadal@chemsys.t.u-tokyo.ac.jp

**Keywords:** cathode, polyanion, high-voltage

## Abstract

High-voltage generation (over 4 V versus Li^+^/Li) of polyanion-positive electrode materials is usually achieved by Ni^3+^/Ni^2+^, Co^3+^/Co^2+^, or V^4+^/V^3+^ redox couples, all of which, however, encounter cost and toxicity issues. In this short review, our recent efforts to utilize alternative abundant and less toxic Fe^3+^/Fe^2+^ and Cr^4+^/Cr^3+^ redox couples are summarized. Most successful examples are alluaudite Na_2_Fe_2_(SO_4_)_3_ (3.8 V versus sodium and hence 4.1 V versus lithium) and *β_1_*-Na_3_Al_2_(PO_4_)_2_F_3_-type Na_3_Cr_2_(PO_4_)_2_F_3_ (4.7 V versus sodium and hence 5.0 V versus lithium), where maximizing Δ*G* by edge-sharing Fe^3+^-Fe^3+^ Coulombic repulsion and the use of the 3d^2^/3d^3^ configuration of Cr^4+^/Cr^3+^ are essential for each case. Possible exploration of new high-voltage cathode materials is also discussed.

## 1. Introduction

Polyanion-positive electrode material for lithium batteries was identified by Delmas, Goodenough, and their co-workers for the NASICON M_2_(XO_4_)_3_ framework in the 1980s [1,2,3]. Later on, Padhi, Nanjundaswamy, and Goodenough discovered a very promising positive electrode material, LiFePO_4_ [4], which is now widely commercialized for stationary use or a power source for electric vehicles. A common advantage of polyanion-type electrodes is their long-term stability of operation due to the rigid structural framework. Additional advantages inherent to LiFePO_4_ that have led to its commercial application are (i) lithium can be extracted at the first charge and functions as a charge carrier, moving back and forward upon charge/discharge, (ii) it can withstand self-decomposition to guarantee a high level of safety, and (iii) it has a suitable operating voltage of 3.4 V versus lithium, which is not so high that it decomposes electrolytes but not too low that energy density is sacrificed [5].

Toward higher voltages, Mn analogue LiMnPO_4_ (4.1 V versus lithium) was investigated but its low electrochemical activity was not acceptable, and this negative feature is common for all Mn-based polyanion-positive electrode materials [4,6,7,8]. Vanadium-based compounds such as LiVPO_4_F [9] operate well at a reasonable voltage range around 4.2 V, but they have been excluded as a commercial option due to the element’s toxicity and the volume change during Li^+^ de/intercalation [10,11]. For an even higher voltage, Co^3+^/Co^2+^ and Ni^3+^/Ni^2+^ redox couples show activity at >4.5 V [12,13,14], but their highly oxidizing nature induces several side reactions unless careful design is applied to both the electrolyte and electrode composite. For a sodium analogue, electrode operation at higher voltages is more important as the Na/Na^+^ potential is ca. 0.3 V higher than the Li/Li^+^ potential. Within the polyanionic materials, strategic design toward high-voltage operation is almost the same in the case of lithium, achieved by introducing V, Co, Ni as a redox center, as represented by Na_4_Co_3_(PO_4_)_2_P_2_O_7_ [15,16,17].

However, the use of V, Co, or Ni is a challenging option for battery engineers as they entail cost and toxicity issues. In particular, for a sodium battery system, a high-voltage system with more abundant and cheap elements would be ideal. In this short review article, after summarizing the influential factors dominating positive electrode voltage, our recent successful attempts to activate Fe^3+^/Fe^2+^ and Cr^4+^/Cr^3+^ redox couples, in Na_2_Fe_2_(SO_4_)_3_ (3.8 V versus sodium and hence 4.1 V versus lithium) [18] and *β_1_*-Na_3_Al_2_(PO_4_)_2_F_3_-type Na_3_Cr_2_(PO_4_)_2_F_3_ (4.7 V versus sodium and hence 5.0 V versus lithium) [19], will be demonstrated.

## 2. Toward Higher Voltage

### 2.1. Inductive Effect in Polyanionic Compounds

The voltage trend of polyanion-based positive electrode materials roughly follows the formal charges of the central atoms in the polyanions, consisting of the idea of the inductive effect [20]. The presence of strong *X*-O covalency stabilizes the antibonding *M*^3+^/*M*^2+^ state through an *M*-O-*X* inductive effect to generate an appropriately high voltage. A series of compounds including large polyanions (*X*O_4_)^y−^ (*X* = S, P, As, Mo, W, *y* = 2 or 3) were explored, and the use of (PO_4_)^3−^ and (SO_4_)^2−^ has been shown to stabilize the structure and lower the *M*^3+^/*M*^2+^ redox energy to useful levels.

### 2.2. Thermodynamic Modification

**Figure 1:**
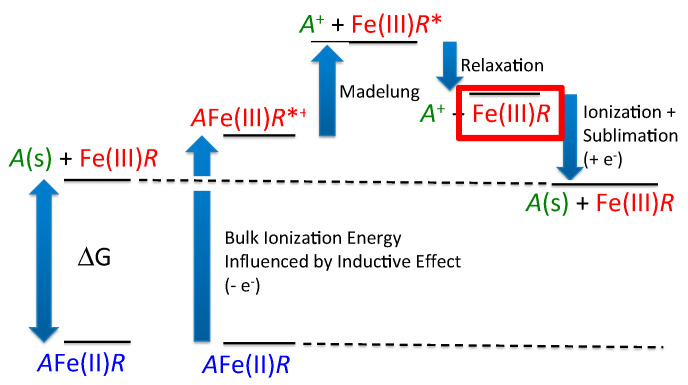
A Born–Haber cycle for the definition of voltage generation in cathode materials based on Fe^3+^/Fe^2+^ redox couple. The notations *R* and R* represent relaxed and unrelaxed frameworks, respectively. Bulk ionization energy, which is closely related to the inductive effect, is an approximation (electronic part) but is not identical to ΔG.

In essence, the voltage is defined as the difference between the lithium chemical potential in the cathode and in the anode, leading to a simple thermodynamic definition, ignoring *PV* and *TS* terms: (*P* = pressure, *V* = volume, *T* = temperature, and *S* = entropy), *E* = (*G*_Li_ + *G*_charged_ − *G*_discharged_)/*nF.* where *G*_Li_, *G*_charged_, and *G*_discharged_, are the Gibbs free energies of lithium metal, charged cathode, and discharged cathode, respectively; *n* is the number of electrons in the redox reaction, and *F* is the Faraday constant. The overall thermodynamic scheme for voltage generation is summarized in Figure 1 based on the Born–Haber cycle [21].

### 2.3. Choice of Transition Metals

**Figure 2:**
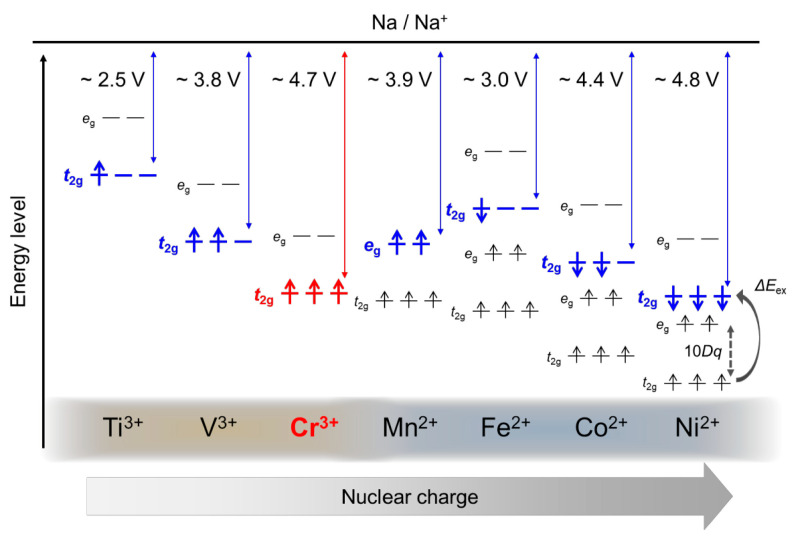
Schematic comparison of operating voltages and d-electron configurations for phosphate-based cathode materials containing 3d transition metals [19]. 10*Dq* and Δ*E*_ex_ indicate an octahedral crystal field splitting energy for 3d orbitals and exchange splitting energy, respectively. Orange and blue shading corresponds to valences of 3 and 2, respectively. Note that there is also a contribution of the Madelung and other energies to the cell voltage that is superimposed on the electronic contribution of the transition metal ions (see Figure 1). Permission is granted by Chemistry of Materials.

Figure 2 shows a schematic derivation of the operation voltages and *d*-band positions of 3*d* transition metal phosphates in sodium ion batteries. [19] In general, a transition metal ion *M^n^*^+^ with a higher atomic number has a deeper valence level owing to a larger effective nuclear charge, resulting in a higher *M*^(*n*+1)+^/*M^n^*^+^ redox potential. Naturally, phosphates with end representatives of the 3d series, such as Co^2+^ and Ni^2+^, typically Na_2_CoPO_4_F and Na_4_*M*_3_(PO_4_)_2_P_2_O_7_ (*M* = Co^2+^ and Ni^2+^), have been reported as high-voltage (4.3 V, 4.4 V, 4.8 V, respectively) cathode materials [16,17,22,23,24]. However, end representatives of the 3d series suffer from energy-level increments either by spin exchange penalty or crystal field splitting. On the other hand, the 3*d*^3^ electron of Cr^3+^ in the *t*_2g_ orbital is free from both spin exchange and crystal field splitting, which can be compensated for the smaller nuclear charge. Indeed, Cr^4+^/Cr^3+^ redox couples in phosphates generate >4.5 V vs. Na/Na^+^ (as presented below) [19], comparable to Co- or Ni-based phosphates.

## 3. High-Voltage System with Fe^3+^/Fe^2+^ and Cr^4+^/Cr^3+^

### 3.1. Pyrophosphates

Of particular interest is the triplite phase of LiFeSO4F [25] and metal-doped Li2FeP2O7 [26,27] possess edge-sharing FeO_6_ octahedra to minimize the Fe-Fe distance, as distinguished from other, lower-voltage Fe-based polyanion electrodes with corner-sharing octahedra. A shorter Fe^3+^–Fe^3+^ distance in the charged state is effective for enlarging *G*_charged_, and hence the operating voltage *E*, while the influence of the discharged state *G*_discharged_ with smaller charge Fe^2+^ can be subordinated in energetics.

**Figure 3:**
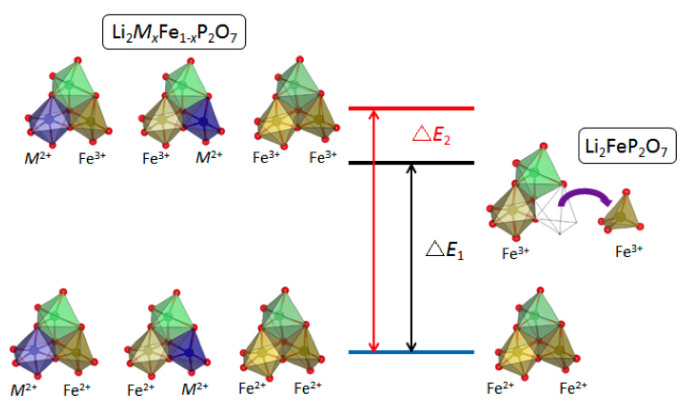
Schematic description of free energy difference between starting and delithiation materials. The right-hand portion is the pristine Li_2_FeP_2_O_7_ system. The spontaneous structural rearrangement (Fe’s migration) destroys the edge-sharing configuration and decreases the free energy of the delithiated state, which results in an energy difference of △*E*_1_. The left-hand portion is the doping system Li_2_*M_x_*Fe_1-*x*_P_2_O_7_. After full delithiation, the Li concentration is higher than that in the Li_2_FeP_2_O_7_ case, because all of the *M* ions remain inert. The remaining Li can block the Fe migration and can stabilize the Fe’s original local structure and the whole crystal structure, which means that the energy difference △*E*_2_ should be higher than △*E*_1_.

The cell voltage for these two materials can reach as high as 3.9 V (vs. Li), which is higher than the value of 3.8 V calculated from the standard redox potentials. The latter has been suggested to be the highest achievable voltage for a Li ion battery utilizing the Fe^3+^/Fe^2+^ redox couple in solid. As shown in Figure 3, the potential tunability for the Fe^3+^/Fe^2+^ redox couple at the unusually high-voltage region of 3.5–3.9 V vs. lithium is similar for any metal *M* doping in the Li_2_*M_x_*Fe_1-*x*_P_2_O_7_ system [27]. The phenomena include two aspects: (1) two redox reactions at different potentials are stabilized with the doping of foreign metal *M*, (2) with more dopant, both of the redox reactions upshift to higher potential, and one even approaches 4 V. Substitution of *M* into Fe sites may suppress the migration of Fe from the FeO_5_ site upon charging, and the two original, distinct Fe sites become robust to stabilize the edge-sharing geometry of FeO_5_ and FeO_6_ polyhedrals with large Fe^3+^-Fe^3+^ coulombic repulsion energy, leading to the two distinct redox reactions with inherently high potentials. The change in the relative energy of the intermediate compounds, which is induced by the unfavorable V_Li_^’^-*M*^2+^(*M*_Fe_^×^) and/or Li_Li_^×^-Fe^3+^(Fe_Fe_•) interaction in the doping case, may be a reason for the further potential upshifting. The classic inductive effect cannot explain the redox potential upshifting phenomenon in this case.

### 3.2. Alluaudites

**Figure 4:**
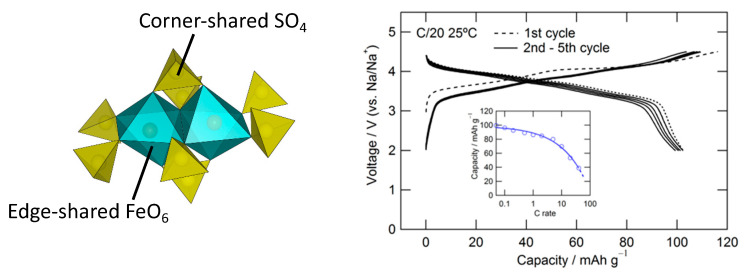
Local coordination structure and charge–discharge voltage profile of Na_2_Fe_2_(SO_4_)_3_.

Compaction of the *M*O_6_ dimer can be more pronounced in an alluaudite framework, where two edge-shared *M*O_6_ octahedra are bridged by a small *X*O_4_ tetrahedron and the *M*-*M* distance becomes much shorter (Figure 4). During the search along the Na_2_SO_4_-FeSO_4_ tie line, we discovered the first sulfate compound with an alluaudite-type framework [18]. Deviating sharply from most of the *A*_x_*M*_2_(*X*O_4_)_3_-type compounds adopting the NASICON-related structures, Na_2_Fe_2_(SO_4_)_3_ does not contain the lantern units [*M*_2_(*X*O_4_)_3_]. It would be convenient to denote *AA’BM*_2_(*X*O_4_)_3_ as general *alluaudite*-type compounds, where *A* = partially occupied Na(2), *A’* = partially occupied Na(3), *B* = Na(1), *M* = Fe^2+^, and *X* = S in the present case.

**Figure 5:**
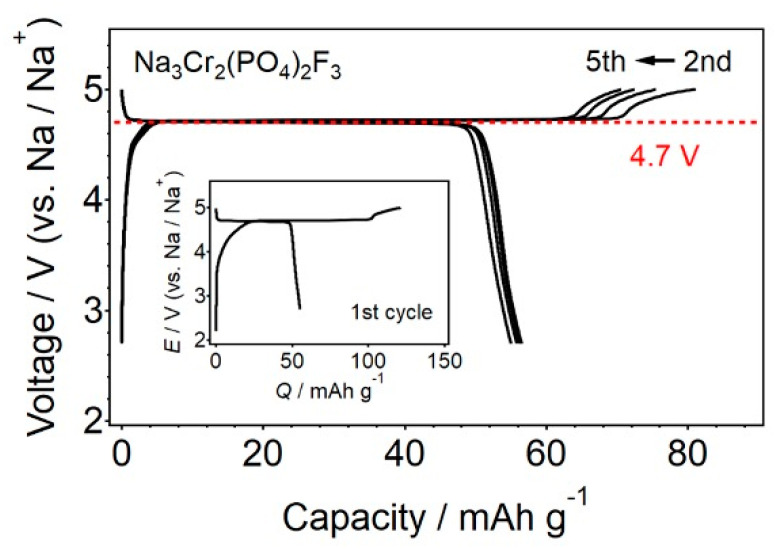
Galvanostatic charge–discharge curves of Na_3_Cr_2_(PO_4_)_2_F_3_ electrode in Na half-cell at a rate of 0.1 C between 2.7 and 5.0 V vs. Na/Na^+^ (1 C = 63.8 mA g^−1^). Inset shows the first cycle.

The Na_2_Fe_2_(SO_4_)_3_ offers an average potential of 3.8 V (vs. Na/Na^+^), with smooth, sloping charge–discharge profiles over a narrow voltage range of 3.3–4.3 V, which is the highest Fe^3+^/Fe^2+^ redox potential obtained in any material environment (Figure 5) [18]. The abnormally high voltage can be explained by the thermodynamic definition of voltage explained in Section 2.2; the edge-sharing geometry of the Fe octahedra in Na_2_Fe_2_(SO_4_)_3_ will raise *G*_charged_ due to the strong Fe^3+^–Fe^3+^ repulsion, leading to high *E*. Additionally, it offers excellent rate kinetics and cycling stability without requiring any additional cathode optimization. It forms an open framework host for the efficient (de)intercalation of Na ions with very low activation energy.

An remarkable feature is that, now, the most commonly accessible redox Fe^3+^/Fe^2+^ can, in principle, generate the high voltage of 3.8 V vs. sodium (and hence 4.1 V vs. lithium). However, the hygroscopicity of the sulphate compounds must be carefully managed.

### 3.3. β_1_-Na_3_Al_2_(PO_4_)_2_F_3_-Type Fluoride Phosphates

Considering the d-level considerations in Section 2.3, an extremely high operating potential of 4.7 V vs. Na/Na^+^ was identified in Na_3__–*x*_Cr_2_(PO_4_)_2_F_3_ (0 < *x* < 1) on account of the Cr^4+^/Cr^3+^ (3d^2^/3d^3^) redox couple, providing a promising design strategy for a high-voltage positive electrode material [19]. Whilst further Na^+^ extraction (*x* > 1) to form NaCr_2_(PO_4_)_2_F_3_ above 5.0 V vs. Na/Na^+^ remains elusive, optimization of the durable cell components for high-voltage operation may enable more activity and greater reversibility. Overall, utilizing low-cost Cr^4+^/Cr^3+^ (3d^2^/3d^3^), instead of Co^3+^/Co^2+^ (3d^6^/3d^7^) or Ni^3+^/Ni^2+^ (3d^7^/3d^8^) as in previously reported polyanion compounds, is worthwhile for the realization of batteries with higher energy density.

## 4. Summary and Perspective

Initiated by Delmas, Goodenough, and co-workers in the 1980s, polyanion-type positive electrode materials now represent a large group of materials for reversible Li^+^, Na^+^, and K^+^ insertion. With a suitable combination of transition metal and framework structure, the operating voltage can be tuned, leading sometimes to a suitable high-voltage range for practical application. Although LiFePO_4_ is the only compound that has been widely applied for commercial use to date, continuous exploration is ongoing in the community toward better batteries with lower cost, high voltage, high safety, and a long calendar life. In addition to the widely examined redox couple based on Fe^3+^/Fe^2+^, Cr^4+^/Cr^3+^ could be an inexpensive yet higher-voltage option for future material development in polyanion compounds.

## Sample Availability

Samples are not available without a certain contract.

## References

[1] Nadiri A., Delmas C., Salmon R., Hagenmuller P. (1984). Chemical and electrochemical alkali metal intercalation in the 3D framework of Fe_2_(MoO_4_)_3_. Rev. Chim. Minérale.

[2] Manthiram A., Goodenough J.B. (1987). Lithium insertion into Fe_2_(MO_4_)_3_ frameworks: Comparison of M = W with M. J. Solid State Chem..

[3] Delmas C., Cherkaoui F., Nadiri A., Hagenmuller P. (1987). A Nasicon-type phase as intercalation electrode: NaTi_2_(PO_4_)_3_. Mat. Res. Bull..

[4] Padhi A.K., Nanjundaswamy K.S., Goodenough J.B. (1997). Phospho-olivines as positive-electrode materials for rechargeable lithium batteries. J. Electrochem. Soc..

[5] Yamada A., Chung S.-C., Hinokuma K. (2001). Optimized LiFePO_4_ for lithium battery cathodes. J. Electrochem. Soc..

[6] Yonemura M., Yamada A., Takei Y., Sonoyama N., Kanno R. (2004). Comparative kinetic study of olivine Li_x_ MPO _4_ (M=Fe,  Mn). J. Electrochem. Soc..

[7] Delacourt C., Laffont L., Bouchet R., Wurm C., Leriche J.-B., Morcrette M., Tarasco J.-M., Masqueliera C. (2005). Toward understanding of electrical limitations (electronic, ionic) in LiMPO_4_ (M = Fe, Mn) electrode materials. J. Electrochem. Soc..

[8] Yamada A., Takei Y., Koizumi H., Sonoyama N., Kanno R., Ito K., Yonemura M., Kamiyama T. (2006). Electrochemical, magnetic, and structural investigation of the li_x_(Mn_y_Fe_1_-_y_)PO_4_ olivine phases. Chem. Mater..

[9] Barker J., Saidi M.Y., Swoyer J.L. (2003). Lithium Iron(II) Phospho-olivines prepared by a novel carbothermal reduction method. Electrochem. Solid State Lett..

[10] Ellis B.L., Ramesh T.N., Davis L.J.M., Goward G.R., Nazar L.F. (2011). Structure and electrochemistry of two-electron redox couples in lithium metal fluorophosphates based on the tavorite structure. Chem. Mater..

[11] Chayambuka K., Mulder G., Danilov D.L., Notten P.H.L. (2020). From Li-ion batteries toward Na-ion chemistries: Challenges and opportunities. Adv. Energy Mater..

[12] Amine K., Yasuda H., Yamachi M. (2000). Olivine LiCoPO_4_ as 4.8 V electrode material for lithium batteries. Electrochem. Solid State Lett..

[13] Wolfenstine J., Allen J. (2005). Ni^3+^/Ni^2+^ redox potential in LiNiPO_4_. J. Power Sources.

[14] Masquelier C., Croguennec L. (2013). Polyanionic (phosphates, silicates, sulfates) frameworks as electrode materials for rechargeable Li (or Na) batteries. J. Am. Chem. Soc..

[15] Bianchini M., Fauth F., Brisset N., Weill F., Suard E., Masquelier C., Croguennec L. (2015). Comprehensive investigation of the Na_3_V_2_(PO_4_)_2_F_3_–NaV_2_(PO_4_)_2_F_3_ system by operando high resolution synchrotron X-ray diffraction. J. Am. Chem. Soc..

[16] Nose M., Nakayama H., Nobuhara K., Yamaguchi H., Nakanishi S., Iba H. (2013). Na_4_Co_3_(PO_4_)_2_P_2_O_7_: A novel storage material for sodium-ion batteries. J. Power Sources.

[17] Gezović A., Vujković M.J., Milović M., Grudić V., Dominko R., Mentus S. (2021). Recent developments of Na_4_M_3_(PO_4_)_2_(P_2_O_7_) as the cathode material for alkaline-ion rechargeable batteries: Challenges and outlook. Energy Storage Mater..

[18] Barpanda P., Oyama G., Nishimura S., Chung S.-C., Yamada A. (2014). A 3.8-V earth-abundant sodium battery electrode. Nat. Commun..

[19] Kawai K., Asakura D., Nishimura S., Yamada A. (2021). 4.7 V Operation of the Cr^4+^/Cr^3+^ redox couple in Na_3_Cr_2_(PO_4_)_2_F_3_. Chem. Mater..

[20] Padhi A.K., Nanjundaswamy K.S., Masquelier C., Okada S., Goodenough J.B. (1997). Effect of structure on the Fe^3+^/Fe^2+^ redox couple in iron phosphates. J. Electrochem. Soc..

[21] Yamada A. (2016). Systematic studies on “abundant” battery materials: Identification and reaction mechanisms. Electrochemistry.

[22] You Y., Manthiram A. (2018). Progress in high-voltage cathode materials for rechargeable sodium-ion batteries. Adv. Energy Mater..

[23] Chayambuka K., Mulder G., Danilov D.L., Notten P.H.L. (2018). Sodium-ion battery materials and electrochemical properties reviewed. Adv. Energy Mater..

[24] Kubota K., Yokoh K., Yabuuchi N., Komaba S. (2014). Na_2_CoPO_4_F as a high-voltage electrode material for Na-ion batteries. Electrochemistry.

[25] Barpanda P., Ati M., Melot B., Rousse G. (2011). A 3.90V iron-based fluorosulphate material for lithium-ion batteries crystallizing in the triplite structure. Nat. Mater..

[26] Furuta N., Nishimura S., Barpanda P., Yamada A. (2012). Fe^3+^/Fe^2+^ redox couple approaching 4 V in Li_2-x_(Fe_1-y_Mny)P_2_O_7_ pyro-phosphate cathodes. Chem. Mater..

[27] Ye T., Barpanda P., Nishimura S., Furuta N., Chung S.-C., Yamada A. (2013). General observation of Fe^3+^/Fe^2+^ redox couple close to 4 V in partially substituted Li_2_FeP_2_O_7_ pyrophosphate solid-solution cathodes. Chem. Mater..

